# Interfacial water molecules at biological membranes: Structural features and role for lateral proton diffusion

**DOI:** 10.1371/journal.pone.0193454

**Published:** 2018-02-23

**Authors:** Trung Hai Nguyen, Chao Zhang, Ewald Weichselbaum, Denis G. Knyazev, Peter Pohl, Paolo Carloni

**Affiliations:** 1 Computational Biomedicine (IAS-5 / INM-9) Forschungszentrum Jülich, Jülich, Germany, RWTH Aachen University, Aachen, Germany; 2 Institute of Biophysics, Johannes Kepler University Linz, Linz, Austria; Oregon State University, UNITED STATES

## Abstract

Proton transport at water/membrane interfaces plays a fundamental role for a myriad of bioenergetic processes. Here we have performed *ab initio* molecular dynamics simulations of proton transfer along two phosphatidylcholine bilayers. As found in previous theoretical studies, the excess proton is preferably located at the water/membrane interface. Further, our simulations indicate that it interacts not only with phosphate head groups, but also with water molecules at the interfaces. Interfacial water molecules turn out to be oriented relative to the lipid bilayers, consistently with experimental evidence. Hence, the specific water-proton interaction may help explain the proton mobility experimentally observed at the membrane interface.

## Introduction

Proton transport between membrane-bound proteins along biological membrane plays a crucial role for bioenergetics of living cells [1–4]. An efficient pathway between protons’ source and sink [3, 5, 6] is represented by proton’s fast and persistent lateral diffusion [7–9]. This process appears to be only weakly dependent of the membrane used [7, 8]: protons move fast along the membrane-water interface prior to being released into the bulk: with the lateral diffusion coefficient in the order of 10^−5^ cm^2^s^-1^ [7–9]. This is similar to the one found in bulk liquid water [7, 10]. The long distance traveled by protons along the membrane [7, 8] is due to a substantial free energy barrier that prevents them from escaping to the bulk [8, 9, 11]. Since its enthalpy component corresponds to the breakage of only a single hydrogen bond, the barrier appears to be mainly entropic [12].

Multistate empirical valence bond (MS-EVB) calculations [13, 14] have provided a microscopic picture of proton transport. They indicated that the excess protons strongly prefer the interface and that the binding affinity of the proton for the surface is mostly driven by the attraction to lipid chemical groups, including phosphate moieties [13, 14]. As a consequence, the proton lateral diffusion coefficient appeared significantly lower than that of the bulk water [13, 14]. Classical molecular dynamics simulations of DMPC lipids with explicit proton (HYDYN) have further suggested that the lateral proton diffusion along the lipids is anomalous and it depends on interfacial water molecules as well [15]. The predicted proton diffusion coefficient turned out to be at least one order of magnitude lower than the experimental value, partially because of limitations of the computational approach used [15].

Here, we re-examine this process by *ab initio* molecular molecular dynamics (MD). To our knowledge, this is the first application of ab initio MD to proton transfer at membrane/water interfaces. *Ab initio* MD simulations have been extensively used to study chemical reactions and proton transfer [16–19]. We perform our simulations on an excess proton at two phospholipids membranes/water interfaces. We find that the lipids’ negatively charged phosphate groups compete for proton binding with electron lone pairs of interfacial water molecules. Water molecules at the interface show a specific orientation, similar to that observed in classical MD simulations [20, 21], pointing to the key role of the proton for water structuring at the interface. Because proton transfer along water molecules is much faster than that along lipid molecules, we propose that the proton/water interactions provide an important ingredient for the experimentally observed fast proton diffusion at the membrane interface.

## Methods

### Systems

**Fig 1:**
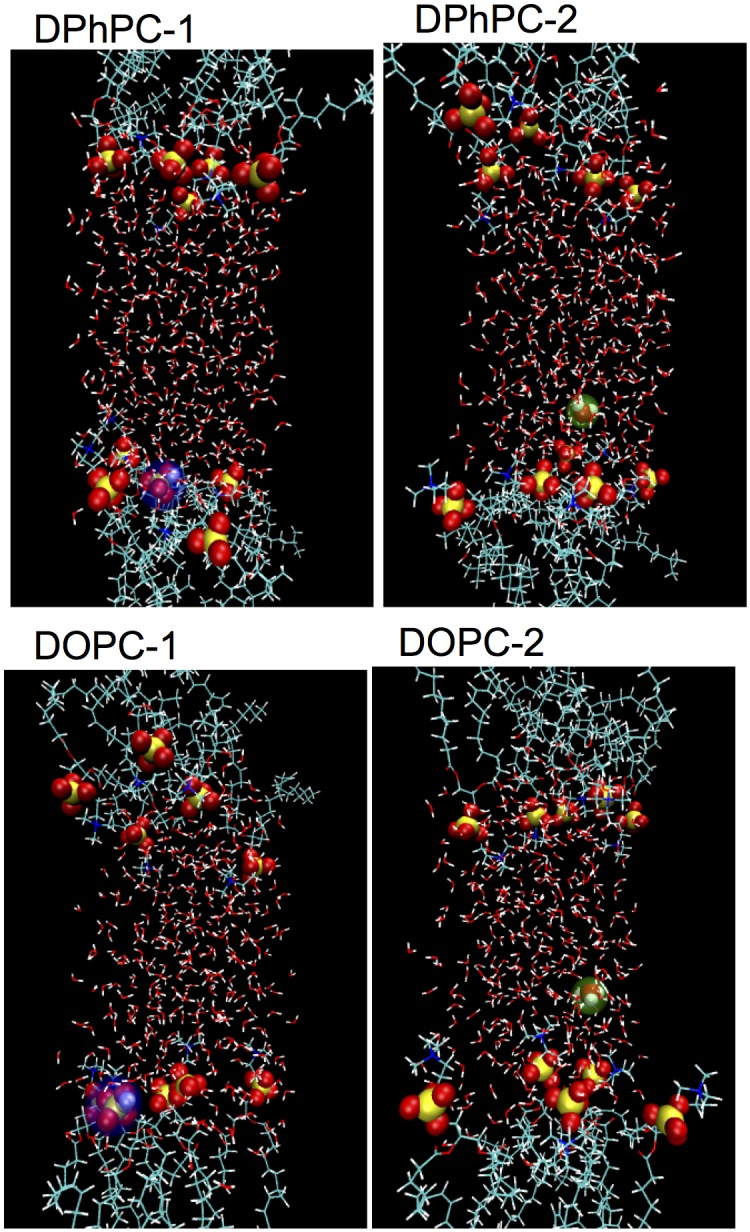
*Ab initio* MD simulation systems. Each system consists of 10 lipids (top: DPhPC and bottom: DOPC), 418 water molecules for DPhPC-1 and DPhPC-2 or 358 water molecules for both DOPC-1 and DOPC-2 water molecules, and one excess proton. Water molecules and the hydrocarbon tails are rendered in sticks. Oxygen is in red, hydrogen in white, carbon in green, nitrogen in blue and phosphorus in yellow. Phosphate groups and the hydronium are depicted as spheres. The protonated phosphate group is highlighted in blue (DPhPC-1, DOPC-1). The interfacial hydronium is highlighted in green (DPhPC-2, DOPC-2).

10 diphytanoylphosphatidylcholine (DPhPC) lipids, 418 water molecules and one proton (2,795 atoms in total) were inserted in a box of edges 19.7 Å × 19.7 Å × 70.6 Å. A previous study on water/hydrophobic liquid interfaces showed that there were two populations of interfacial proton, one is attached to the surface and relatively immobile while the other stays in the interfacial water layer and very mobile [11]. Therefore, in this work we initiated the simulations with two possible positions of the proton. The proton was either attached to the phosphate’s oxygen atom of a lipid (referred to as DPhPC-1 in Fig 1) or placed in the interfacial water layer, about three water molecules away from the nearest phosphate group (referred to as DPhPC-2 in Fig 1).

Ten 1,2-dioleoyl-sn-glycero-3-phosphocholine (DOPC) molecules, 358 water molecules and one proton (2,455 atoms in total) were inserted in a box of edges 19.1 Å × 19.1 Å × 69.8 Å. Similarly, the proton was either attached to the phosphate’s oxygen atom of a lipid (referred to as DOPC-1 in Fig 1) or placed in the water layer near the lipid surface (referred to as DOPC-2 in Fig 1).

Each system without the excess proton underwent 20 ns classical MD simulations at T = 310 K and P = 1 atm. The NAMD program [22] was employed. Because of the availability, the CHARMM36 force field [23, 24] was used for DOPC lipids, the AMBER one for DPhPC. The AMBER the force field was constructed following the standard Amber procedure [25, 26]. GAFF force field and RESP charges were derived by the electrostatic potential obtained through B3LYP/6-31G(d,p) single point calculations. The force field parameters are reported in Table A1 in S1 File.

We used the TIP3P model [27] for water molecules. Periodic boundary conditions were imposed on the simulation box. The temperature was controlled at 310 K by the Langevin thermostat [28] while the pressure was kept at 1 atm using the Noseé-Hoover Langevin piston barostat [29]. Long-range electrostatic interactions were evaluated using the Particle Mesh Ewald (PME) method [30]. The cutoff for the real part of the PME and for the van der Waals interaction was set to 10 Å. An integration time step of 2 fs was used.

The area per lipid was close to the experimental values [31, 32] at the end of the simulations (Table A2 in S1 File). The last snapshots from classical MD simulations were subject to subsequent *ab initio* MD simulations after adding the excess proton as described above.

### *Ab initio* MD

In CPMD simulations, the electronic degrees of freedom are treated with quantum mechanics. Because nuclear quantum effects for the proton transfer in liquid water are rather small [33], we treated all of the nuclei (including the excess proton) as classical particles that obey Newtonian mechanics. The quantum electronic structure problem was solved within the framework of the density functional theory (DFT), using Becke-Lee-Yang-Parr (BLYP) functional [34, 35] as implemented in the CPMD 3.15.3 [36]. An empirical van der Waals correction [37] was applied to improve the description of liquid water in *ab initio* MD simulation [38] as a tradeoff between computational efficiency and chemical accuracy. This choice of the exchange-correlation functional and van der Waals correction has been largely investigated by some of us in previous studies involving aqueous interfaces and lipids [11, 39]. The electronic wavefunction was expanded in a plane wave basis set with a cutoff of 70 Ry. A time step of 0.097 fs and a fictitious electron mass of 400 au were used. First, several annealing/heating runs were carried out to equilibrate the structures. Then, 2 ps-long constant volume and temperature (NVT) *ab initio* MD simulations were performed, in which the temperature was controlled at 310 K by using a Nosé-Hoover chain thermostat [40]. Finally, the last snapshots underwent constant volume and energy (NVE) MD simulations for 10 ps (DPhPC-1 and DOPC-1), 9 ps (DPhPC-2) and 12 ps (DOPC-2).

### Analysis

#### The joint probability of proton transfer events

**Fig 2:**
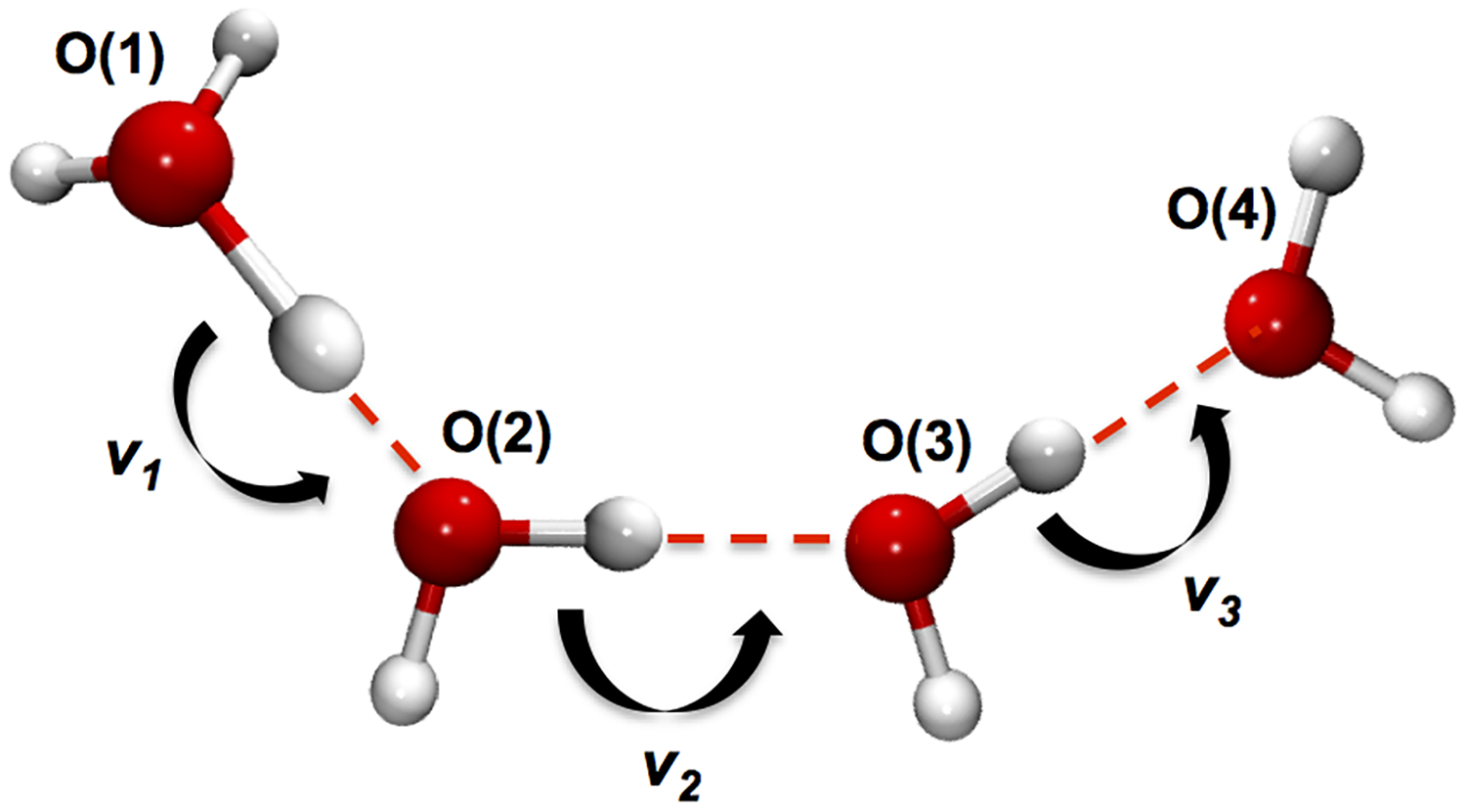
Schematic of three consecutive proton transfer coordinates. Oxygen ad hydrogen atoms are shown as red and white spheres, respectively. Hydrogen bonds are represented as dashed lines. *v*_*1*_, *v*_*2*_ and *v*_*3*_ are the proton transfer coordinates of Eq (1).

This quantity is calculated as follows. First, the proton transfer coordinates are defined as in Hassanali *et al*. [19] (Fig 2). The first coordinate, *v*_1_, is defined as the difference between the distance of the proton to the first oxygen atom O(1) (*r*_O(1)-H_) and the distance of the same proton to the second oxygen O(2) (*r*_O(2)-H_):

$$v_{1}=r_{O(1)-H}-r_{O(2)-H}.$$ (1)

The second and the third coordinate, *v*_2_ and *v*_3_, are defined in a similar manner for pairs of oxygen atoms O(2), O(3) and O(3), O(4), respectively.

The joint probability between two proton transfer coordinates *v*_1_ and *v*_2_ is then calculated as [19]:

$$P\left(v_{1},v_{2}\right)=\frac{1}{N_{f}}\sum_{i=1}^{N_{w}}\delta_{v_{1},v_{1}^{i}}\cdot \delta_{v_{2},v_{2}^{i}},$$ (2)

where i runs over the number of all water wires that house the proton (*N*_*f*_) counted along the MD trajectories. $\delta_{v_{1},v_{1}^{i}}=1$ if $v_{1}^{i}\in \left[v_{1}-\Delta v,v_{1}+\Delta v\right)$ and $\delta_{v_{1},v_{1}^{i}}=0$ otherwise, where Δ*ν* = 0.03 is the bin size. The joint probability associated to triple consecutive proton transfer events *v*_1_, *v*_2_ and *v*_3_ is calculated as *P*(*ν*_1_,(*ν*_2_ + *ν*_3_)/2) as in Hassanali *et al*. [19]

#### The orientation distribution of water molecules at a given distance from the instantaneous water/membrane interface

This quantity reads [41]:

$$P\left(\text{cos}(\theta ),Z\right)=\frac{1}{\rho_{bulk}L^{2}}\langle \underset{i}{\sum }\delta \left(\text{cos}(\theta_{i})-\text{cos}(\theta )\right)m\delta \left(z_{i}-Z\right)\rangle .$$ (3)

**Fig 3:**
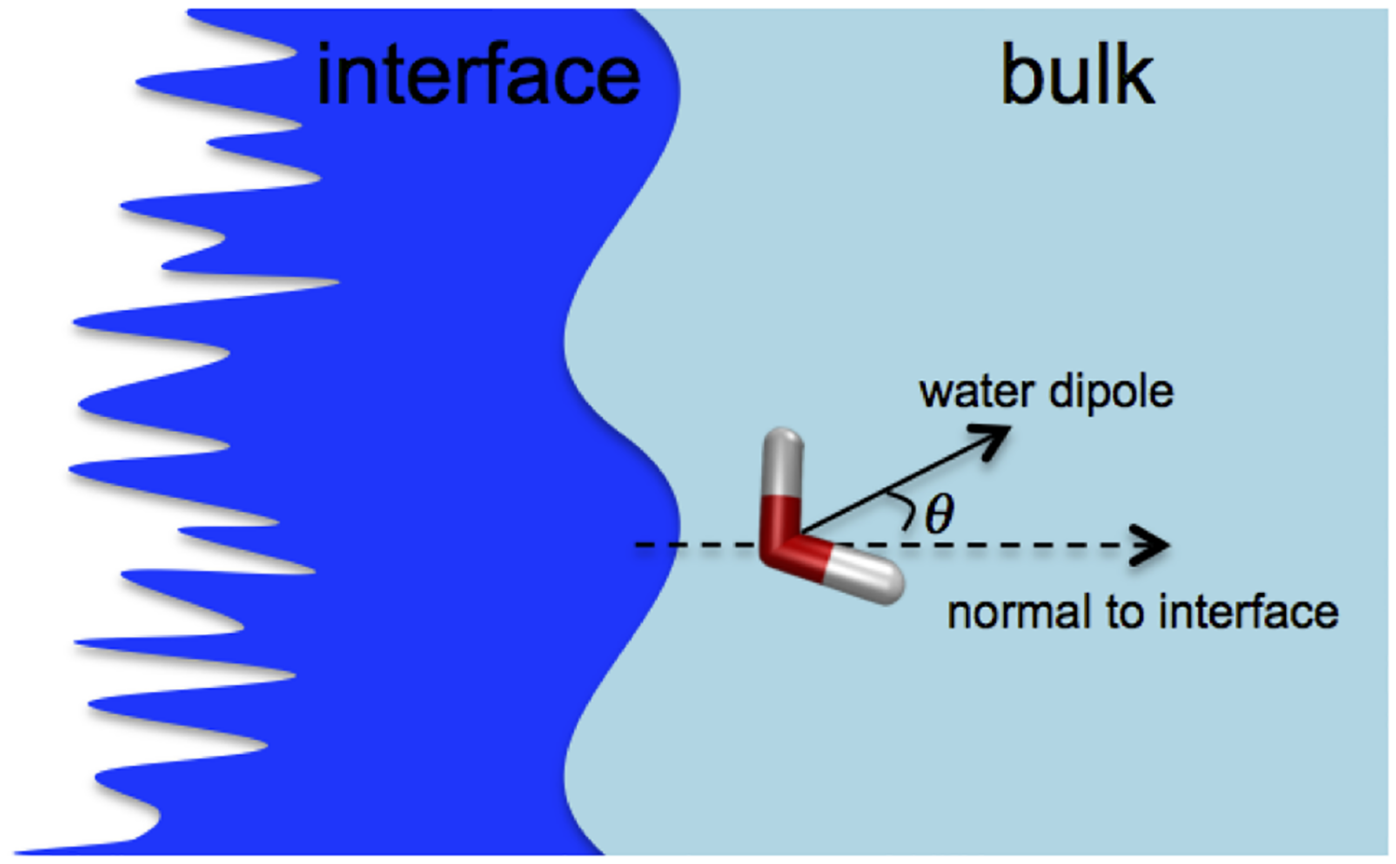
Schematic of orientation angle of water molecule with respect to the interface.

*Z* is the distance from the Willard-Chandler instantaneous interface [42]. The sum runs over all the water molecules. *L* is the size of the box in the direction parallel to the surface. *θ*_*i*_ is the angle formed by the dipole moment of water molecule *i* and the normal to the interface at point ${\vec{s}}_{i}^{*}$, which is the nearest point on the surface $\vec{s}(t)$ to water molecule *i* (see Fig 3 for the schematic representation of the orientation angle). *m* is the mass of a water molecule. *z*_*i*_ is the smallest distance between water molecule *i* and the surface. *ρ*_*bulk*_ is the water density in the bulk.

## Results and discussion

### Proton transfer

The excess proton was initially attached either to a phosphate’s oxygen (DPhPC-1 and DOPC-1) or to interfacial water molecules (DPhPC-2 and DOPC-2). In the first two simulations, the excess proton remained with the two nearby phosphate groups (one of them was where the proton was originally attached) for the entire duration (about 10 ps). It moved back and forth between the two nearby phosphate groups via one or two bridging water molecules. This indicates that the excess proton prefers water-wire transport mechanism over the lipid protonable group hopping mechanism.

**Fig 4:**
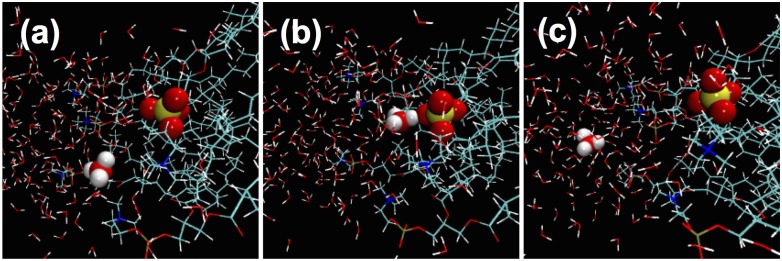
Proton motion observed in the *ab initio* MD simulations of DPhPC-2 system. The snapshots are taken at 1 ps when the excess proton is > 6 Å from its nearest phosphate (a); at 4 ps when the excess proton is < 3 Å from the phosphate (b) and at 7 ps when the excess proton is again > 6 Å away from the phosphate (c). Atom color scheme is the same as in Fig 1. The hydronium and phosphate group are represented by spheres. The rest of the system is shown in stick representation. The other leaflet of the lipid bilayer is not shown for the sake of clarity.

In DPhPC-2, the proton stayed in the interfacial region for about 2 ps (panel a in Fig 4). Then it moved to the proximity of a phosphate group and stayed there until about 5 ps (panel b in Fig 4). Finally, the proton migrated back to the interfacial water and remained there until the end of the simulation, about 9 ps (panel c in Fig 4). This back-and-forth shuffling of the excess proton between the proximity of the phosphate groups and the interfacial water layers indicates high proton mobility. This delocalization of the excess proton at the membrane surfaces supports previous theoretical studies [13–15].

**Fig 5:**
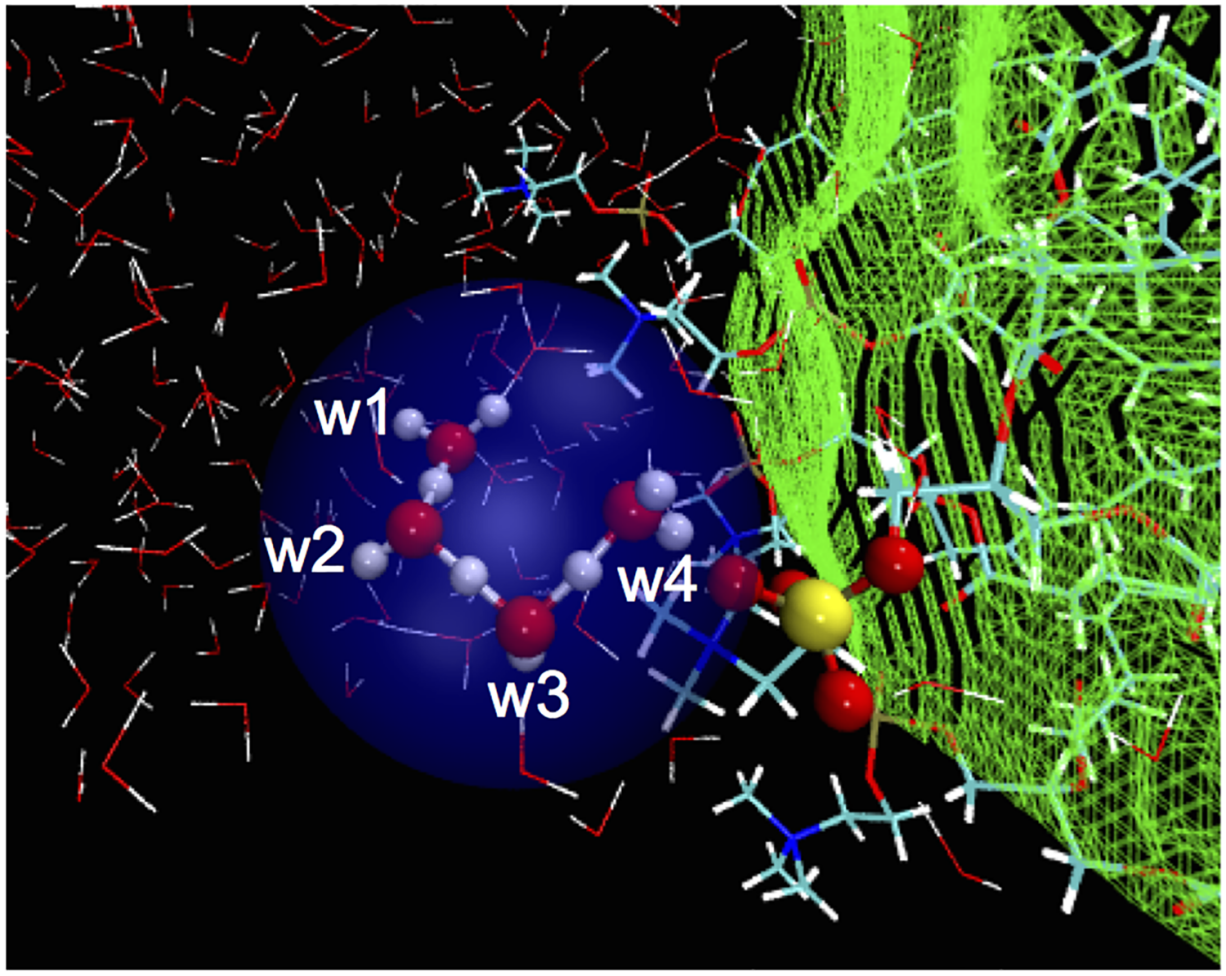
Concerted proton transfer from the interfacial water to a phosphate group. This is observed after about 9 ps in the *ab initio* MD simulation of DOPC-2 system. Atom color scheme is the same as in Fig 1. The water wire utilized is highlighted in blue. The proton-accepting phosphate moiety is spheres. Other water and lipid molecules are shown as sticks. Note W1 to W4 correspond to O(1) to O(4) in Fig 2.

**Fig 6:**
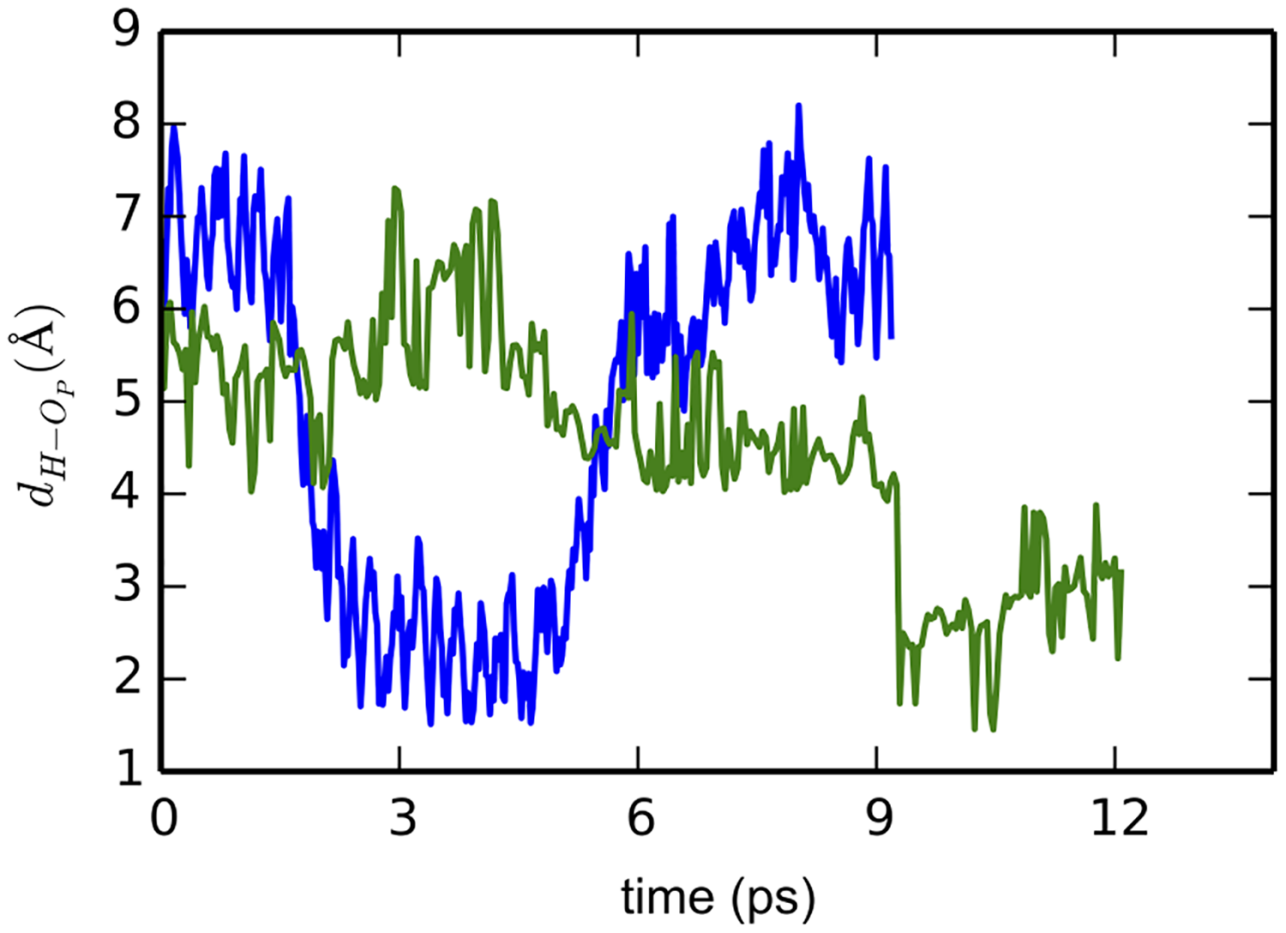
Distance from the excess proton to the nearest phosphate oxygen as a function of simulation time.

In DOPC-2, the excess proton diffused in the interfacial water layer among nearby water molecules via Grotthuss mechanism. The distance to the proton to the interface did not change significantly, indicating that the diffusion is mostly lateral. It remained in the interfacial water layer until about 9 ps. Then, it quickly migrated towards a phosphate group, jumping simultaneously over 3 consecutive hydrogen bonds (Fig 5). The proton stays close to the phosphate until the end of our simulation. The attraction of proton from water’s lone pairs competes with the phosphate groups (Fig 5) and this competition may lead to the excess proton being quite mobile near the membrane (Fig 6). However, we did not see any protonation of the phosphate groups in the time-scale accessed by our *ab initio* MD simulations. It is worth to note that Fig 6 does not imply that there are protonation events between the excess proton and phosphate group. The minimum distance in Fig 6 is about 2 Å. This value is much longer than the distance of an O-H covalent bond. In fact, due to electrostatic attraction, we observed movements of the excess proton towards the phosphate group, but no chemical bond was formed.

**Fig 7:**
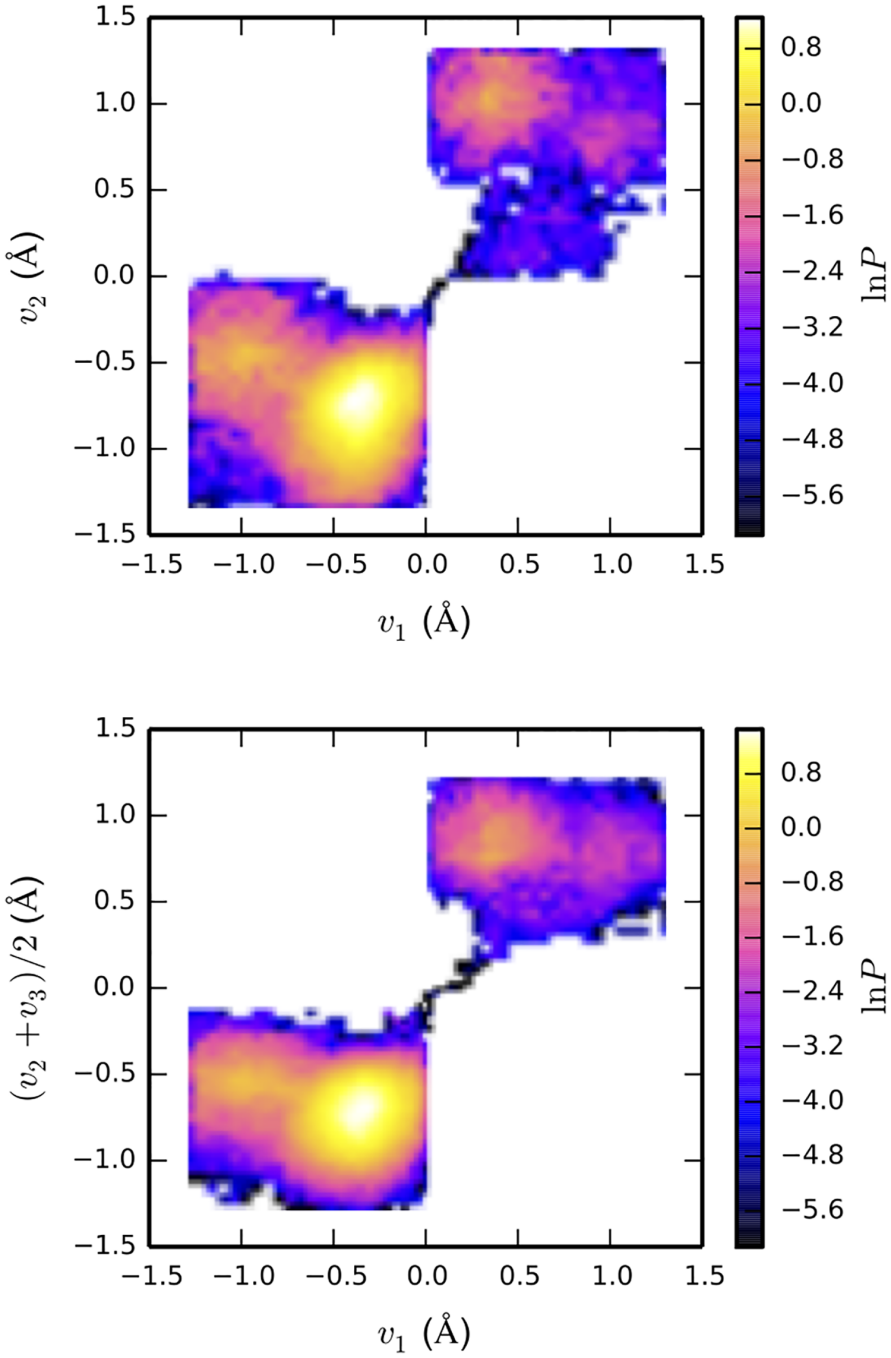
2D histograms of proton transfer coordinates of DOPC-2 from *ab intio* MD simulations. Note that the color bar is on a logarithmic scale. See Methods section and Fig 2 and for the definition of proton transfer coordinates.

The concerted proton transfer at the water/membrane interface in DOPC-2 is evident from Fig 7, which shows the 2D histograms of proton transfer coordinates defined in Fig 2. In the upper panel of Fig 7 the joint probability being nonzero at *v*_1_ = 0 and *v*_2_ = 0 indicates that the protons simultaneously appear in the middle of both O(1)-O(2) and O(2)-O(3) bonds (see Fig 2 for the schematic representation of the water wire). Similarly, in the bottom panel of Fig 7 the joint probability being nonzero at *v*_1_ = 0 and (*v*_2_ + *v*_3_)/2 = 0 implies that the protons simultaneously appear in the middle of three consecutive bonds O(1)-O(2), O(2)-O(3) and O(3)-O(4). Similarly, concerted proton transfer has been previously observed in an *ab initio* MD study of a proton in bulk water [9, 19] and reported for the lateral proton transfer at water/air interfaces [43]. Subsequently, the proton stayed close to the phosphate until the end of the simulation (about 12 ps). Conceivably, it did not return to the interfacial waters due to the short time scale of our *ab initio* MD simulation. For both systems DPhPC-2 and DOPC2, we found that the proton mainly interacts with the phosphate but not the carbonyl group. We did not observe simultaneous jumping of the excess proton over multiple water molecules in DPhPC-2 system. This is probably due to the short time scale of *ab initio* MD simulations.

### Orientation of water molecules

**Fig 8:**
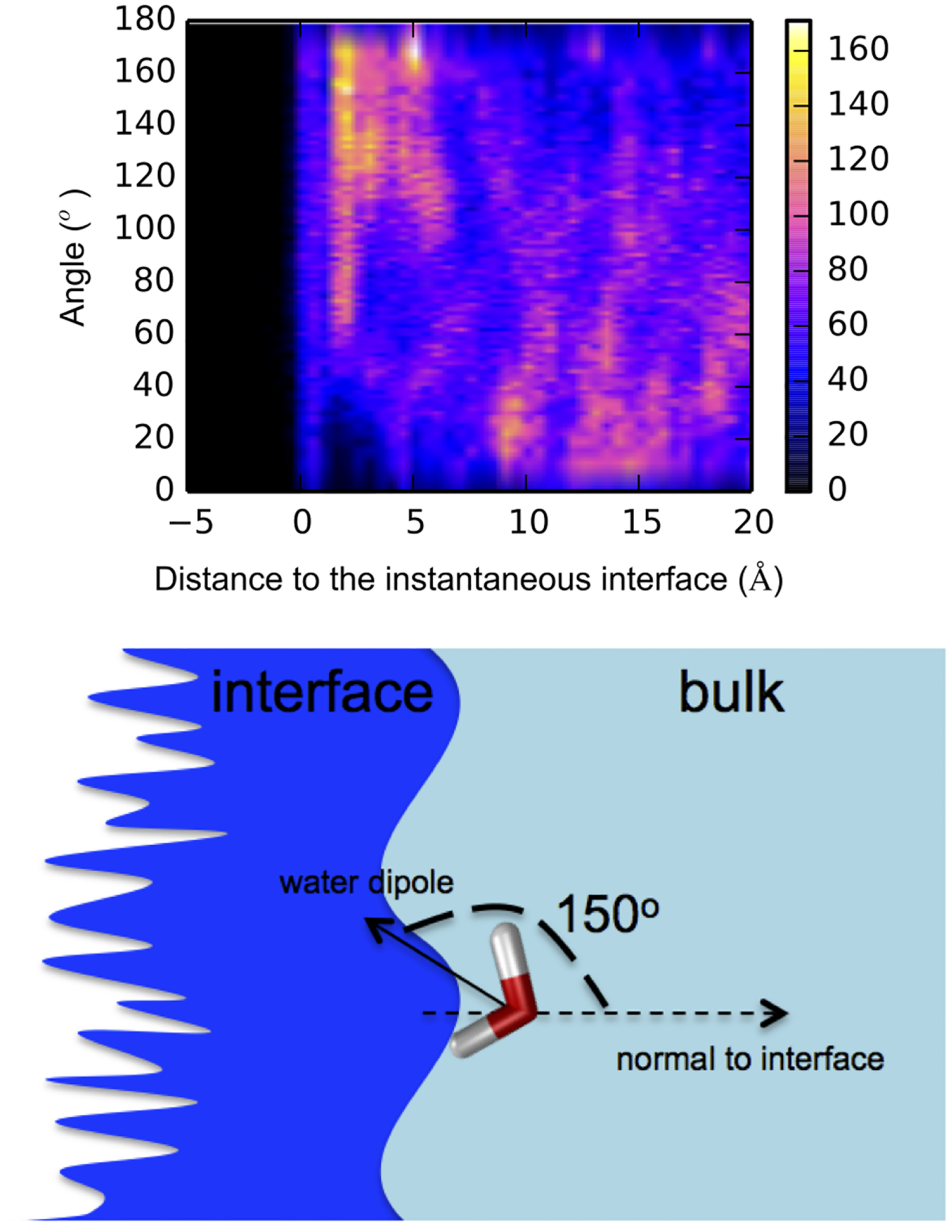
Top: 2D histogram of the angle between water dipole moment and interface normal, and the distance from the instantaneous water/membrane interface for DOPC-2 system. Trajectories were collected using *ab initio* MD simulations; Bottom: Schematic representation of approximately 150° orientation of water molecule with respect to the interface.

Here we consider orientation of interfacial water with respect to the instantaneous surface. The roughness of the instantaneous surface is rather large, as shown by the green mesh grid in Fig 5. It expands for one to two layers of water molecules in water/membrane systems, as seen in the water orientation plot in Fig 8 upper panel and Figure A1 in S1 File. The relaxation time of the overall surface can be in the order of tens of picoseconds [42]. The water dipole moments in the first two layers (< 6 Å) near the interface are oriented at about 150° with respect the normal to the instantaneous interface (Fig 8 upper panel). Indeed, the water wire mediating the concerted proton transfer (W3 and W4 in Fig 5) are oriented at 139° and 157°, respectively. This water arrangement, in which more hydrogen bonds point towards the surface (Fig 8, lower panel) are also found for the rest of simulations system (DOPC-1, DPhPC-1 and DPhPC-2, see Fig A1 in S1 File). Such a common interfacial water hydrogen-bonding pattern is likely to facilitate fast proton transport from interfacial water molecules to the phosphate groups. The water orientation found here is compatible with the dominant orientation of about 120° reported in classical molecular dynamics simulation studies for water molecules at lipid/water interfaces [20, 21], where a preferred orientation has been observed qualitatively [20].

These results are consistent with phase-sensitive vibrational sum frequency generation spectroscopy [44] and measurements of membrane dipole potential [45–47]. The specific orientation of the interfacial water layer found here may facilitate proton movement along the membrane.

### Limitations

**Fig 9:**
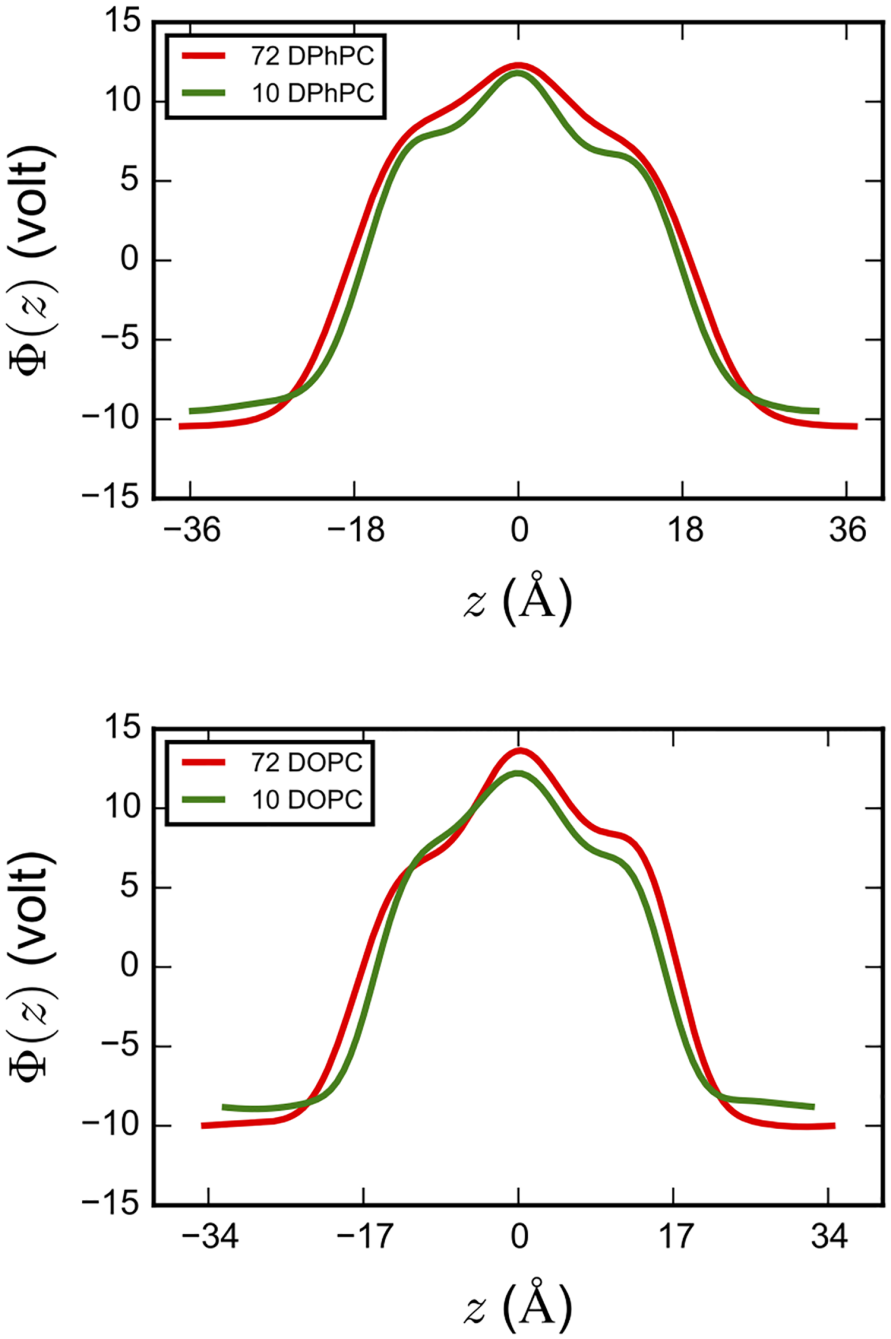
Electrostatic profile in the direction *z* perpendicular to the interface of DPhPC (top) and DOPC (bottom) lipids from classical MD simulations. *z* = 0 corresponds to the center of the lipid bilayers.

As any modeling study, this work has limitations. In particular, here finite size effects might play a role considering the small supercell of our *ab initio* MD simulations that contains 10 lipid molecules. To investigate this issue, we have calculated the electrostatic potential for the classical MD simulation boxes without the excess proton (see text in S1 File for more details). Fig 9 compares the electrostatic potential profile in the direction perpendicular to the interface in our system of 10 lipids to a water/membrane system containing as many as 72 lipids, using classical MD simulations. The overall shape of the electrostatic profiles of the small and large systems is essentially the same. This demonstrates that the electrostatic environment is relatively insensitive to the simulation system size. Despite of the relatively small water slab used in our *ab initio* MD simulations; the density plateau of the bulk water is reproduced as well (see Fig A2 in S1 File).

Second, the short time scale of *ab intio* MD simulation did not allow us to sample the full dynamics of lipid bilayers, therefore, the conclusion of this work reflects only the local chemical environment at the water/membrane interface. Similarly, our simulations may be biased by the initial condition in the atomic configurations because of the short time scale.

Finally, there is always the issue regarding the intermolecular potential in which its accuracy is determined by the exchange-correlation functional used in simulations, although BLYP plus the empirical dispersion correction is known to give a rather reasonable description of bulk liquid water [38] and has been used to studied membrane systems [39].

## Conclusions

We have presented an ab initio MD study on an excess proton at DOPC and DPhPC membrane interfaces. Our calculations suggest that the excess proton is preferentially located at the interface, in line with previous MS-EVB [13] and HYDYN [15] studies. The excess proton is quite mobile in spite of its strong interactions with the membrane because of the competing attraction of both the negatively charged phosphate groups and the lone electron pairs of interfacial water molecules. The water molecules are oriented differently from those of bulk water. Due to their preferable orientation, the water molecules at the membrane interface play a key role for proton transport. This finding might help to explain why titratable residues are not required for proton migration along membranes [48].

## Supporting information

S1 File Supporting text, figures and tables. (PDF)
