# Supplementary material for: Interfacial water molecules at biological membranes: Structural features and role for lateral proton diffusion
Source: PLoS One. 2018 Feb 23;13(2):e0193454. doi: 10.1371/journal.pone.0193454 (PMC5825111; doi:10.1371/journal.pone.0193454)
Supplement: S1 File — (PDF) [file pone.0193454.s001.pdf]

Supporting Information

Interfacial water molecules at biological membranes: structural features and role for lateral proton diffusion

Trung Hai Nguyen[1,#a], Chao Zhang[1*,#b], Ewald Weichselbaum[2], Denis G. Knyazev[2], Peter Pohl[2], Paolo Carloni[1*]

[1] Computational Biomedicine (IAS-5 / INM-9) Forschungszentrum Jülich, 52425 Jülich, Germany, RWTH Aachen University, 52056, Aachen, Germany

[2] Institute of Biophysics, Johannes Kepler University Linz, 4040, Linz, Austria

[#a] Current Address: Department of Chemistry, Illinois Institute of Technology, Chicago, IL 60616, USA

[#b] Current Address: Department of Chemistry, Ångström Laboratory, Lägerhyddsvägen 1, BOX 538, 75121 Uppsala, Sweden

* Corresponding author

E-mail: chao.zhang@kemi.uu.se (CZ), p.carloni@fz-juelich.de (PC)

MD simulations of systems containing 72 lipids

The simulation boxes contained 72 lipid molecules and 4209 and 4203 water molecules for DOPC and DPhPC respectively. The initial box edges were 51 Å x 51 Å x 70 Å for DOPC system and 53 Å x 53 Å x 70 Å for DPhPC system. The systems underwent 5 ns long NPT equilibration and subsequently 20 ns long NVT production runs using the protocol described in the main text.

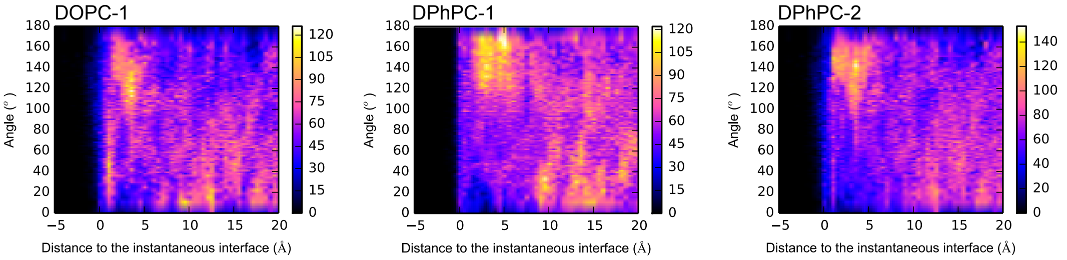

Fig A1. 2D histograms of the angle between water dipole moment and interface normal, and the distance from the instantaneous water/membrane interface for DOPC-1, DPhPC-1 and DPhPC-2 system.

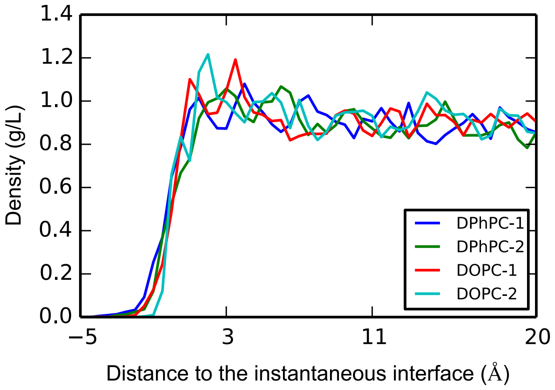

Fig A2. Water density as a function of distance from the instantaneous interface.

Table A1. AMBER force field parameters for DPhPC.

Atom names	GAFF Atom types	REST charges
C24	c3	-0.304792
H43, H44, H45	hc	0.061803
C23	c3	0.385375
C28	c3	-0.304792
H55, H56, H57	hc	0.061803
H42	hc	-0.064478
C22	c3	-0.178588
H40, H41	hc	0.032934
C21	c3	0.057404
H38, H39	hc	-0.008206
C20	c3	-0.078449
H36, H37	hc	0.009786
C19	c3	0.246699
C27	c3	-0.265526
H52, H53, H54	hc	0.055100
H35	hc	-0.038141
C18	c3	-0.187307
H33, H34	c3	0.024728
C17	c3	0.203433
H31, H32	hc	-0.031490
C16	c3	-0.151684
H29, H30	hc	0.022385
C15	c3	0.239158
C26	c3	-0.257038
H49, H50, H51	hc	0.050504
H28	hc	-0.041279
C14	c3	-0.089245
H26, H27	hc	0.017833
C13	c3	0.019435
H24, H25	hc	-0.000535
C12	c3	-0.125538
H22, H23	hc	0.017471
C11	c3	0.447449
C25	c3	-0.363647
H46, H47, H48	hc	0.074660
H21	hc	-0.078203
C10	c3	-0.367876
H19, H20	hc	0.077043
C9	c	0.923101
O5	o	-0.639492
O6	os	-0.484254
C6	c3	0.049027
H15, H16	h1	0.073431
C7	c3	0.319067
C8	c3	0.143494
O4	os	-0.532524

P1	p5	1.456447
O1	o	-0.855013
O2	os	-0.579664
C1	c3	0.350553
C2	c3	-0.015061
N1	n4	0.192988
C3	c3	-0.238559
H6, H7, H8	hx	0.130876
C4	c3	-0.238559
H9, H10, H11	hx	0.130876
C5	c3	-0.238559
H12, H13, H14	hx	0.130876
H4, H5	hx	0.073389
H2, H3	h1	0.005753
O3	o	-0.855013
H17, H18	h1	0.056468
H1	h1	0.046723
O8	os	-0.539589
C29	c	0.958201
O7	o	-0.663825
C30	c3	-0.287227
H58, H59	hc	0.049996
C31	c3	0.356739
C45	c3	-0.373219
H85, H86, H87	hc	0.082002
H60	hc	-0.037018
C32	c3	-0.155705
H61, H62	hc	0.031669
C33	c3	0.003572
H63, H64	hc	-0.005336
C34	c3	-0.040275
H65, H66	hc	0.001334
C35	c3	0.244224
C46	c3	-0.290624
H88, H89, H90	hc	0.058846
H67	hc	-0.045075
C36	c3	-0.125198
H68, H69	hc	0.012073
C37	c3	0.177683
H70, H71	hc	-0.023401
C38	c3	-0.236317
H72, H73	hc	0.047095
C39	c3	0.282269
C47	c3	-0.295880
H91, H92, H93	hc	0.060597
H74	hc	-0.045560
C40	c3	-0.076044
H75, H76	hc	0.007880
C41	c3	0.055186
H77, H78	hc	-0.012015
C42	c3	-0.188069
H79, H80	hc	0.037842

C43	c3	0.433539
C48	c3	-0.340052
H94, H95, H96	hc	0.068223
H81	hc	-0.078181
C44	c3	-0.340052
H82, H83, H84	hc	0.068223

Table A2. Comparison of area per lipid (Å^2) between MD simulations and experiment for DOPC (1) and DPhPC (2).

Systems	Experiment	MD Simulation
DOPC	72	73
DPhPC	80	77

References

1. Poger D, Mark AE. On the Validation of Molecular Dynamics Simulations of Saturated and cis-Monounsaturated Phosphatidylcholine Lipid Bilayers: A Comparison with Experiment. J Chem Theory Comput. 2009;6(1):325-36.
2. Tristram-Nagle S, Kim DJ, Akhunzada N, Kucerka N, Mathai JC, Katsaras J, et al. Structure and water permeability of fully hydrated diphytanoylPC. Chem Phys Lipids. 2010;163(6):630-7.
